# In vivo visualization of lipophagy dynamics using the tfLiveDrop reporter mice

**DOI:** 10.1016/j.jlr.2026.101033

**Published:** 2026-04-02

**Authors:** Siqiao Gong, Qiaofei Zhang, Hongluan Wu, Xiaocui Chen, Lijing Liu, Yongming Chen, ZeSen Feng, Shangmei Li, Hongyong Su, Jiansong Qi, Jixin Tang, Zhennan Ye, Chen Yang, Huafeng Liu

**Affiliations:** National Clinical Key Specialty Construction Program (2023), Guangdong Provincial Key Laboratory of Autophagy and Major Chronic Non-communicable Diseases, Key Laboratory of Prevention and Management of Chronic Kidney Disease of Zhanjiang City, Department of Nephrology, Institute of Nephrology, Affiliated Hospital of Guangdong Medical University, Zhanjiang, China

**Keywords:** lipid droplets, lipophagy, type 2 diabetes, kidney development, LiveDrop

## Abstract

Lipophagy, a selective form of autophagy, is critical for maintaining cellular lipid homeostasis. However, understanding its dynamic regulation and pathophysiological significance in vivo has been hindered by a lack of sensitive and versatile monitoring tools. To address this gap, we generated the *tfLiveDrop* (mCherry-eGFP-LiveDrop) reporter mouse by integrating a tandem mCherry-eGFP fluorescent probe with the lipid droplet-targeting domain of glycerol-3-phosphate acyltransferase 4 (GPAT4, the rate-limiting enzyme in triacylglycerol synthesis), termed the LiveDrop domain. This model enables real-time, spatiotemporal visualization of lipophagic flux at single-cell resolution in living animals. We initially validated the sensitivity and specificity of the tfLiveDrop reporter in primary renal tubular epithelial cells (TECs). Systemic mapping of lipophagic activity across organs revealed pronounced heterogeneity in basal lipophagic activity under physiological conditions. Furthermore, in a model of type 2 diabetes, we demonstrated that lipophagic flux is dysregulated in a tissue-specific manner in male mice, underscoring its pivotal role in disease-associated lipid metabolism. Notably, longitudinal tracking during kidney development uncovered a programmed wave of lipophagic activity that is essential for lipid homeostasis during renal maturation. Our findings provide a powerful and versatile platform for in vivo lipophagy research, establishing a foundation for elucidating its functional contributions to metabolic disorders and organ development.

Lipid droplets (LDs) are ubiquitous, dynamic organelles central to cellular lipid storage and metabolism (1, 2). Their regulated formation and degradation are critical for maintaining lipid homeostasis, and dysregulation of these processes is implicated in a spectrum of metabolic, inflammatory, and neurologic diseases (3, 4, 5). A key pathway for LDs degradation is lipophagy, a selective form of autophagy. This process involves the recognition and engulfment of LDs by phagophores, leading to the formation of autophagosomes. These vesicles then fuse with lysosomes, where LD components are hydrolyzed by acidic hydrolases into free fatty acid (FFA) for metabolic utilization (6). Through this mechanism, lipophagy serves as an essential regulator of energy balance and cellular function in major metabolic tissues (7, 8).

Despite its physiological and pathological significance (9, 10, 11), our understanding of lipophagy in vivo remains rudimentary, primarily due to a lack of tools to dynamically monitor this process in living organisms. Current gold-standard methods, such as electron microscopy, merely provide static snapshots and are not amenable to probing the dynamics of lipophagic flux within complex tissues (12). While immunofluorescence assays can indicate proximity between LDs and autolysosomes, they cannot reliably distinguish selective lipophagy from nonselective autophagic capture, nor can they conclusively demonstrate functional flux (13, 14). Thus, there is a pressing need for a sensitive, specific, and genetically encodable reporter that can visualize and quantify lipophagic flux in real time and at cellular resolution in vivo.

The recent success of tandem fluorescent protein reporters in mapping mitophagy and ER-phagy (the selective clearance of mitochondria and endoplasmic reticulum) in vivo has demonstrated the transformative power of such tools for studying organelle-selective autophagy (15, 16). Inspired by this approach, we sought to develop an analogous reporter for lipophagy. Here, we present the generation and validation of the tfLiveDrop reporter mouse, which targets a pH-sensitive dual-fluorescent tag (mCherry-eGFP) to the LD surface via the LiveDrop domain. This model enables direct, real-time visualization of lipophagic flux via differential pH stability of the fluorophores.

The tfLiveDrop model bridges a critical methodological gap. We demonstrate its utility by mapping basal lipophagic activity across tissues, revealing its dysregulation in metabolic disease, and uncovering a previously unknown, developmentally programmed wave of lipophagic flux essential for renal maturation. This tool provides a versatile, robust platform to fundamentally advance in vivo studies of lipophagy in health, disease, and development.

## Materials and Methods

### Animals

The CAG-LSL*-mCherry-eGFP-LiveDrop* (*tfLiveDrop*) conditional reporter mouse was generated by GemPharmatech Co., Ltd., using a targeted knock-in strategy at the *Rosa26* locus. The construct consists of a ubiquitously expressed CAG promoter, followed by a *loxP*-flanked STOP cassette, and the open reading frame (ORF) encoding the mCherry-eGFP-LiveDrop fusion protein (preceded by a Kozak sequence). This design ensures that reporter expression depends solely on Cre-mediated excision of the STOP cassette. To achieve whole-body reporter activation, tfLiveDrop mice were crossed with Tg(pCAG-iCre) mice (GemPharmatech Co., Ltd.), which express an improved Cre *(iCre)* recombinase under the control of a modified CAG promoter, resulting in widespread recombination.

Genotyping was performed by polymerase chain reaction (PCR) using genomic DNA extracted from tail biopsies. The following primer sets were used to distinguish between alleles: *Cre*: Forward, 5′-TTCGGCTTCTGGCGTGTGA-3′, Reverse 5′-CTGACTTCATCAGAGGTGGCATC-3′; *tfLiveDrop* knock-in allele: Forward 5′-GCCTTTATGCCTTTAATCCCAGC-3′, Reverse 5′-TGGCGTTACTATGGGAACATACGTC-3′; *tfLiveDrop* wild-type (WT) allele: Forward 5′-AAATGTAGGGCCAGAGTTTAGCCA-3′, Reverse 5′-TGGAAATCAGGCTGCAAATCTC-3′. All animal procedures were performed in compliance with the guidelines approved by the Institutional Animal Care and Use Committee (IACUC) of the Affiliated Hospital of Guangdong Medical University (Protocol No. AHGDMU-LAC-B-202312-0021).

### Induction of the type 2 diabetes (T2D) mouse model

A well-established model of T2D was induced in mice using a combined protocol of high-fat diet (HFD) feeding and low-dose streptozotocin (STZ) administration, as previously described (17). Briefly, 8-week-old male *CAG-Cre; tfLiveDrop*^*+/−*^ mice were randomly divided into two groups. The model group was fed an HFD (60% kcal from fat; diet code XTHF60; Synergy Bio) for 4 weeks to induce insulin resistance, whereas the control group received a standard chow diet. After the dietary intervention, mice in the model group were fasted overnight and then administered STZ (Sigma-Aldrich, S0130) intraperitoneally (i.p.) at 50 mg/kg body weight for five consecutive days. The STZ was freshly dissolved in 0.1 M sodium citrate buffer (pH 4.5). Control mice received only the citrate buffer vehicle. Following the STZ injections, the model mice remained on the HFD for an additional 12 weeks to consolidate the diabetic phenotype. At the experimental endpoint, mice were fasted for 6 h and anesthetized, after which blood and tissue samples were collected.

### Induction of short-term lipid accumulation

To visualize lipophagic flux in various organs of female mice, 8-week-old female *CAG-Cre;tfLiveDrop*^*+/−*^ mice were randomly assigned to two groups. The experimental group was fed an HFD (60% kcal from fat; XTHF60) for 3 weeks to induce systemic lipid loading and initiate LD formation, while the control group received a standard chow diet.

### Tissue sample collection and processing

Mice (n = 5–10 per group) were euthanized by an overdose of sodium pentobarbital. Terminal blood was collected immediately via cardiac puncture. To remove residual blood from the tissue vasculature, systemic perfusion was performed via the left ventricle with ice-cold phosphate-buffered saline (PBS) until the effluent ran clear. Following perfusion, the target organs (kidney, liver, heart, small intestine, lung, and brain) were rapidly dissected and processed for subsequent analysis. For cryosectioning, tissues were fixed in 4% paraformaldehyde (PFA) at 4°C for 2 h, cryoprotected by immersion in 30% (w/v) sucrose in PBS at 4°C overnight, embedded in Optimal Cutting Temperature (OCT) compound (Sakura Finetek), and stored at −80°C until sectioning. For paraffin embedding, tissues were fixed in 4% neutral-buffered formalin at room temperature for 24 h, then processed through a graded ethanol series, cleared in xylene, and embedded in paraffin blocks.

### Histopathological analysis of tissues

Tissue sections of 3 μm thickness were cut using a microtome and mounted onto glass slides. For histological evaluation, the sections were stained with hematoxylin and eosin (H&E) using a commercial kit (Solarbio, G1120) following the manufacturer's protocol. Brightfield images of the stained sections were acquired using a microscope (Olympus BX53) equipped with a digital camera (Olympus DP74) under consistent lighting conditions.

### Quantification of serum lipid profiles

Serum triglyceride (TG) and total cholesterol (TC) levels were measured using corresponding commercial kits (Jiancheng Bio, Nanjing, China; TG, A110-1-1; TC, A111-1-1) according to the manufacturer's instructions. Briefly, serum samples were mixed with the provided working reagents and incubated at 37°C for 10 min. The absorbance of the reaction mixture was measured at 500 nm using a microplate spectrophotometer (BioTek ELX800). All assays were performed in duplicate to ensure data reliability.

### Oil Red O staining

Oil Red O staining was performed to visualize neutral lipids. A working solution was freshly prepared by dissolving 30 mg of Oil Red O powder (Sigma-Aldrich, O0625) in 6 ml of absolute isopropanol with continuous shaking at 37°C for 30 min. Distilled water (4 ml) was then added to achieve a final concentration of 60% isopropanol. The solution was equilibrated at room temperature for 10 min and centrifuged at 12,000 × g for 2 min to remove insoluble precipitates. For staining, 6-μm-thick frozen tissue sections were incubated with the freshly prepared supernatant for 15 min at room temperature. Sections were briefly differentiated in 60% isopropanol for 1 min, thoroughly rinsed with distilled water for 5 min. Finally, counterstained with hematoxylin (Solarbio, G1080) and mounted with an aqueous mounting medium (Boster, AR1018).

### Cell culture and transfection

Mouse tubular epithelial cells (mTECs) (provided by Dr Jeffrey B. Kopp, NIH), were cultured in Dulbecco's Modified Eagle Medium (DMEM; Gibco, C11995500BT) supplemented with 10% fetal bovine serum (FBS; Gibco, 10,270,106) and 1% penicillin-streptomycin (Pen-Strep; Gibco, 15,140,122). Cells were maintained at 37°C in a humidified incubator with 5% CO_2_. For transient expression, mTECs were seeded into 6-well plates (Corning, 3,516). At approximately 70–80% confluence, cells were transfected with 2.5 μg of the CAG-LSL-*mCherry-eGFP-LiveDrop*-Wpre-PolyA plasmid construct using Lipofectamine 3,000 reagent (Invitrogen, L3000015), following the manufacturer's protocol. For stable cell line generation, stable *Atg5*-knockout (KO) mTECs were previously generated in our laboratory using CRISPR/Cas9 with the following sgRNA sequence: AAGATGTGCTTCGAGATGTG. To construct the lipophagy reporter, the *mCherry-eGFP-LiveDrop* sequence was PCR-amplified from the *tfLiveDrop* targeting vector and subcloned into the pCDH-EF1-MCS-IRES-puro lentiviral plasmid via XbaI and BamHI sites. Lentiviral particles were harvested and used to transduce both WT and *Atg5*-KO mTECs.

Primary renal TECs were isolated from *CAG-Cre;tfLiveDrop* mice using a previously established enzymatic digestion method (18). In brief, renal cortical tissues were dissected and minced into approximately 1 mm^3^ fragments. Fragments were then digested in PBS (Gibco, 14,190,144) containing 1 mg/ml collagenase IV (Sigma-Aldrich, C5138) at 37°C for 10 min with gentle agitation. Digestion reaction was terminated by adding DMEM/F12 medium (Gibco, 11,320,033) containing 10% FBS. The cell suspension was sequentially filtered through 70 μm and 40 μm cell strainers (Corning, 352,350, 352,340) to remove undigested tissue. After centrifugation at 300 × g for 5 min, the cell pellet was resuspended in complete DMEM/F12 medium, supplemented with 10% FBS and 1% Pen-Strep. Finally, the isolated Primary renal TECs were seeded onto chamber slides for subsequent experimental treatments and analysis.

### Western blot analysis

Total cell lysates were prepared in RIPA lysis buffer (Beyotime, P0013) supplemented with a protease inhibitor cocktail (Beyotime, ST506) and a phosphatase inhibitor (Applygen, P1260). Protein concentrations were determined using a BCA Protein Assay Kit (Thermo Fisher Scientific, 23,227). Equal amounts of protein (15–20 μg per lane) were separated by 12% SDS-PAGE and transferred onto PVDF membranes, followed by immunoblotting with special primary antibodies: ATG5 (Abcam, ab108327; 1:2000), LC3 (Abcam, ab51520; 1:2000), SQSTM1/p62 (Abcam, ab56416; 1:2000), and β-Actin (Proteintech, 66009-1-Ig; 1:5000) overnight at 4 °C. Then incubated with HRP-conjugated secondary antibodies (Bio-Rad, 1,706,515/1,706,516; 1:5000) for 1 h at room temperature. Protein bands were visualized using Clarity Western ECL Substrate (Bio-Rad, 1,705,061) and imaged on an Azure Biosystems C500 Imaging System. Band intensity quantification was performed using ImageJ software (NIH).

### In vitro LD formation and lipophagy monitoring

To induce LD formation, cells were incubated with a 0.1/0.2 mM FFA mixture (oleic acid: palmitic acid = 2:1; Siduorui Biotechnology) in complete DMEM for 24 h. Control cells received an equivalent volume of 10% fatty acid-free BSA.

To monitor lipophagic flux in real time, primary renal TECs expressing the mCherry-eGFP-LiveDrop reporter were pulsed with 0.2 mM FFA for 24 h to induce LD loading, followed by a 12 h chase in serum-free medium to stimulate catabolic lipophagy. Thirty minutes prior to imaging, the cells were stained with 50 nM LysoTracker Deep Red (Thermo Fisher Scientific, L12492) in DMEM. Time-lapse images were captured using an Olympus FV3000 confocal microscope with a 60x oil-immersion objective.

To functionally dissect the lipophagic pathway, cells were treated with specific pharmacological modulators during the chase period: 5 mM 3-methyladenine (3-MA; Selleck, S2767), an inhibitor of autophagy initiation, or 20 nM bafilomycin A1 (BafA1; Selleck, S1413), which impairs lysosomal acidification. These inhibitors were added during the last 6 h of the chase period.

### Lipid-Deep Red/lipid blue staining

To confirm the colocalization of the tfLiveDrop reporter signal with LDs, Primary renal TECs isolated from mice or *Atg5*-KO stable expressing mCherry-eGFP-LiveDrop mTECs were stained with 1 μM Lipid-Deep Red (Dojindo Molecular Technologies, LD04)/100 nM Lipid Blue (AIE Institute, AIE-LD-B01) in the dark at 37°C for 30 min. Finally, the samples were mounted in an appropriate medium for confocal microscopy imaging.

### Immunofluorescence staining

For fluorescence microscopy, fixed cells or 5-μm-thick frozen tissue sections were prepared. Samples were fixed with 4% paraformaldehyde (PFA) in PBS for 10 min at room temperature, permeabilized with 0.2% Triton X-100 in PBS for 10 min, and blocked with 5% BSA for 1 h at room temperature. Subsequently, samples were incubated overnight at 4°C with the primary antibodies: anti-PLIN2 (Proteintech, 15294-1-AP; 1:500), anti-LAMP1 (HUABIO, HA722827; 1:500). After washing three times with PBS (5 min per wash), samples were incubated with an Alexa Fluor 647-conjugated anti-rabbit secondary antibody (Thermo Fisher Scientific, A-31573; 1:300) for 1 h at room temperature in the dark. Nuclei were counterstained with DAPI (Beyotime, C1005) for 10 min. Finally, the samples were mounted with Fluoro-Gel medium (Electron Microscopy Sciences, 17,985-41) and images were acquired using an Olympus FV3000 confocal microscope. For co-localization analysis, line scan profiling was performed using ImageJ software.

### Immunohistochemistry staining

Paraffin-embedded tissues were sectioned at a thickness of 3 μm, deparaffinized in xylene, and rehydrated through a graded ethanol series. Antigen retrieval was performed by heating the sections in citrate buffer (Beyotime, P0081) for 15 min. Endogenous peroxidase activity was blocked with 3% H_2_O_2_ for 15 min, and non-specific binding sites were blocked with 5% BSA for 1 h at room temperature. Sections were incubated overnight at 4 °C with anti-PLIN2 (Proteintech, 15294-1-AP; 1:500) primary antibody. After washing three times with PBS, sections were incubated with HRP-conjugated secondary antibodies (Bio-Rad, 1,706,515; 1:300) for 1 h at room temperature. Staining was developed using an AEC substrate kit (Zsbio, ZLI-9036), and the sections were counterstained with hematoxylin for 2 min. Finally, the stained sections were mounted with an aqueous medium and imaged using an Olympus BX43 light microscope. Staining intensity was quantified using ImageJ (NIH).

### Statistical analysis

Statistical analyses were performed using GraphPad Prism 10. Data are presented as mean ± standard deviation (SD) from at least three independent experiments. Comparisons between two experimental groups were performed using an unpaired Student's *t* test. For multiple group comparisons, one-way analysis of variance (ANOVA) was performed, followed by Dunnett's post hoc test. A *P* value < 0.05 was considered statistically significant. A statistical chart was generated using GraphPad Prism 10.

## Results

### Generation and validation of tfLiveDrop reporter mice for monitoring lipophagic flux

**Fig. 1:**
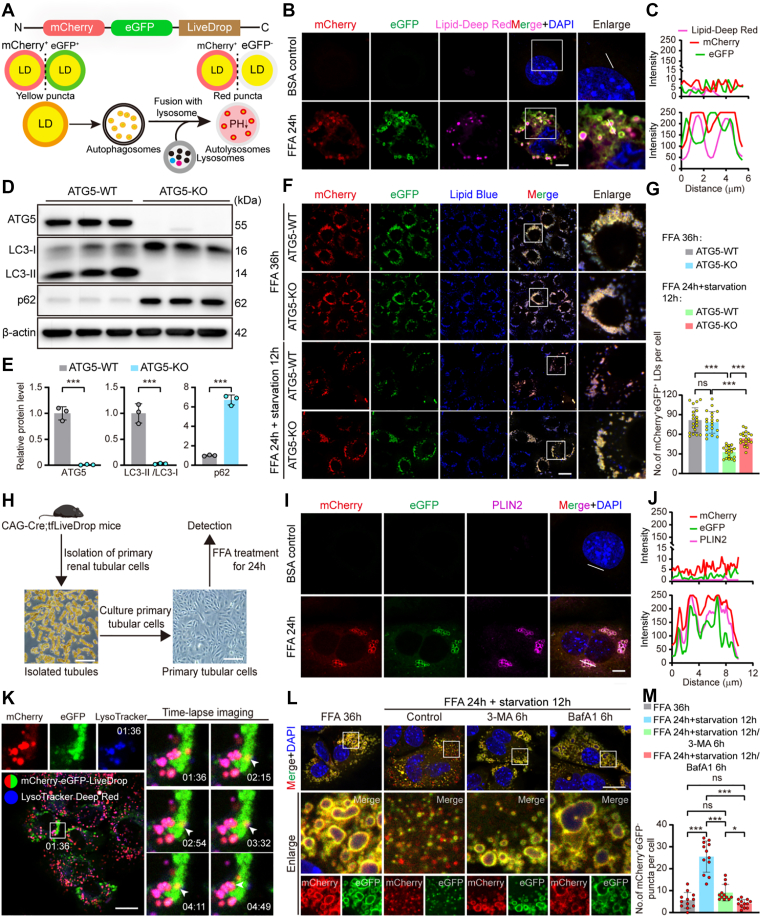
Design and validation of the tfLiveDrop reporter system for monitoring lipophagic flux. A: Schematic illustration of the fluorescent signal conversion principle during lipophagy. LDs exhibit yellow puncta (mCherry^+^GFP^+^) in the cytosol or when sequestered by autophagosomes; upon fusion with lysosomes, the acidic environment quenches eGFP fluorescence, resulting in mCherry-only puncta. B: *Confocal microscopy images* of tfLiveDrop signals colocalizing with Lipid-Deep Red-stained LDs in mTECs transfected with the reporter construct. *Upper*: BSA control; *lower*: 0.1 mM FFA treatment. Scale bar: 10 μm. C: Plot profile shows the relative fluorescence intensities of mCherry, eGFP, and Lipid-Deep Red along the indicated path. D, E: Validation of Atg5 knockout efficiency. Western blot analysis (D) and corresponding quantification (E) showing the expression levels of ATG5, p62, and LC3 in WT and *Atg5*-KO mTECs. F: *Confocal microscopy images* of tfLiveDrop signals in WT and *Atg5*-KO mTECs. Images were captured under both basal conditions and starvation-induced lipophagy. Scale bar: 10 μm. G: Quantification of mCherry^+^eGFP^+^ LDs per cell in each group (n = 20 cells per group). H: Experimental timeline: Primary renal TECs were isolated from male *CAG-Cre*; *tfLiveDrop*^+/−^ mice and treated with 0.1 mM FFA for 24 h. I: Confocal microscopy images of colocalization of tfLiveDrop signals with the LD coat protein PLIN2 in Primary renal TECs. Scale bar: 10 μm. J: Plot profile showing the relative fluorescence intensities of mCherry, eGFP, and PLIN2 along the indicated path. K: Time-lapse live-cell imaging of primary renal TECs. Cells were pulsed with 0.2 mM FFA for 24 h and chased in starvation medium. The *white arrowheads* indicate the dynamic transition of a yellow punctum to a mCherry^+^ only signal within a lysosome (LysoTracker Deep Red, blue) (see Supplemental Movie S1). L: *Confocal microscopy images* of tfLiveDrop signals in primary renal TECs, Cells were subjected to a 0.2 mM 24 h FFA pulse followed by a 12 h starvation chase. Autophagy was modulated by the addition of 5 mM 3-MA (inhibitor of autophagosome nucleation) or 20 nM Bafilomycin A1 (inhibitor of lysosomal acidification) at the 6 h midpoint of the chase period. Scale bar: 10 μm. M: Quantification of mCherry^+^eGFP^-^ puncta per cell in each group (n = 12 cells per group). Data are shown as mean ± SD; ns, not significant, ∗*P* < 0.05, ∗∗*P* < 0.01, ∗∗∗*P* < 0.001; Student's *t* test for (E), and one-way ANOVA for (G, M).

To enable real-time in vivo monitoring of lipophagy, we engineered a tandem fluorescent reporter mouse model, termed tfLiveDrop. This system utilizes a pH-sensitive dual-fluorophore probe (mCherry-eGFP) fused to the LD-targeting domain of GPAT4 (19). Under neutral pH conditions (LDs in the cytosol or sequestered by autophagosomes), the probe emits yellow fluorescence (mCherry^+^eGFP^+^). Upon delivery to acidic lysosomes via lipophagy, the eGFP signal is quenched while mCherry remains stable, generating red-only puncta (mCherry^+^eGFP^-^). Thus, the mCherry^+^eGFP^-^ signal serves as a direct, quantifiable readout of lipophagic flux (Fig. 1A). We first validated the specific LD targeting of the tfLiveDrop construct in vitro. Transient transfection of the tfLiveDrop (CAG-LSL-*mCherry-eGFP-LiveDrop*-Wpre-PolyA) expression construct into mTECs, followed by FFA stimulation to induce LD formation, resulted in precise colocalization of the mCherry/eGFP signals with Lipid-Deep Red^+^ LDs, confirming correct targeting (Fig. 1B, C). Having established the LD-specific localization of the reporter, we next sought to determine whether the observed mCherry^+^eGFP^+^ to mCherry^+^eGFP^-^ conversion indeed reflects autophagy-dependent LD degradation. To this end, we generated Atg5-knockout (KO) mTECs stably expressing the tfLiveDrop reporter. ATG5 deletion had no impact on FFA-induced LD formation, indicating that the initial biogenesis of LDs remained intact. However, upon FFA withdrawal to induce nutrient deprivation, *Atg5*-KO cells exhibited a marked accumulation of mCherry^+^eGFP^+^ LDs compared to WT cells, indicating that delivery of LDs to lysosomes was significantly impaired. These results suggest that tfLiveDrop-mediated lipid clearance is an autophagy-dependent process (Fig. 1D–G).

To generate the reporter mouse line, we utilized CRISPR/Cas9 to knock in a single copy of a conditional expression cassette (CAG-LSL-*mCherry-eGFP-LiveDrop*-Wpre-PolyA) into the *Rosa26* safe-harbor locus of C57BL/6 mice. This cassette is designed to express the tfLiveDrop probe (mCherry-eGFP fused to the LiveDrop domain) in a Cre-dependent manner. The LiveDrop domain ensures robust anchoring to LDs upon recombination. This founder line was designated “*tfLiveDrop*-floxed” (Supplemental Fig. S1A). To achieve systemic reporter expression, we crossed tfLiveDrop-floxed mice with mice expressing Cre recombinase ubiquitously, generating *CAG-Cre; tfLiveDrop* reporter mice (Supplemental Fig. S1B). Successful germline transmission and the expected genotypes were confirmed by PCR analysis of tail genomic DNA (Supplemental Fig. S1C).

We next verified reporter functionality in primary renal TECs isolated from *CAG-Cre;tfLiveDrop* male mice (Fig. 1H). Under basal conditions, cells exhibited negligible fluorescence (Fig. 1I), consistent with low physiological LD levels and indicating minimal cellular perturbation by the probe (20, 21). Upon FFA stimulation to induce LD formation, the mCherry and eGFP signals robustly colocalized with the endogenous LD coat protein PLIN2 (Fig. 1I, J), recapitulating the specific targeting observed in vitro (Fig. 1B). Long-term live-cell imaging following FFA stimulation and subsequent chase in serum-free medium revealed the dynamic progression of lipophagy: mCherry^+^eGFP^+^ puncta were initially observed in close apposition to LysoTracker Deep Red-labeled lysosomes, followed by gradual loss of eGFP fluorescence upon fusion with acidic lysosomes, resulting in the formation of mCherry^+^eGFP^-^ puncta. This transition directly visualizes the stepwise delivery of lipid droplets to lysosomes and their subsequent degradation (Fig. 1K, Supplemental Movie S1). In contrast, inhibiting autophagy initiation with 3-MA or impairing lysosomal acidification with BafA1 resulted in the aberrant accumulation of mCherry^+^eGFP^+^ LDs, with BafA1 treatment leading to a more pronounced accumulation than 3-MA (Fig. 1L, M). These findings demonstrate that the tfLiveDrop system serves as a sensitive and quantitative platform for real-time monitoring of lipophagic flux.

**Fig. 2:**
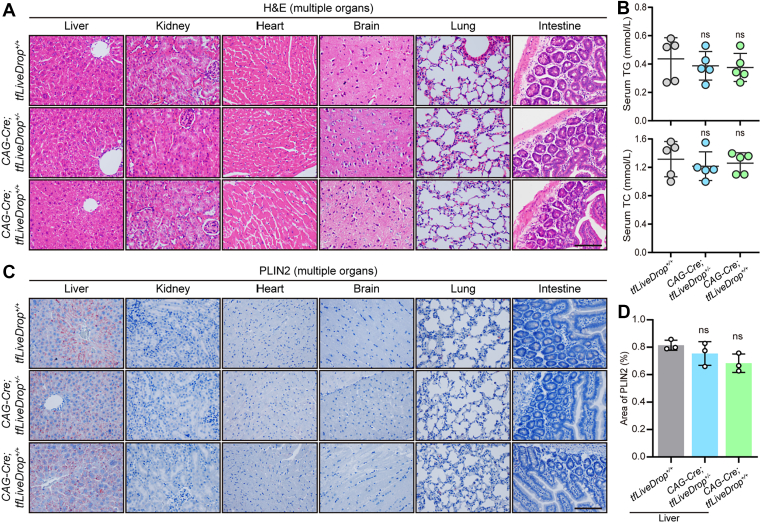
tfLiveDrop expression does not alter basal lipid homeostasis or tissue morphology. A: Representative H&E staining of major organs from male *tfLiveDrop*^+/+^, *CAG-Cre*; *tfLiveDrop*^+/^^−^, and *CAG-Cre*; *tfLiveDrop*^+/+^ mice under basal conditions. Scale bars: 50 μm. B: Serum TG and TC levels of *tfLiveDrop*^+/+^,*CAG-Cre;tfLiveDrop*^+^^/−^,*CAG-Cre;tfLiveDrop*^+/+^ mice under basal condition (n = 5). C: Representative images of PLIN2 immunostaining in tissue sections of systemic organs from the indicated genotypes. Scale bars: 50 μm. D: Quantification of PLIN2^+^ areas (Each data point represents the mean value from 15 fields per mouse, n = 3). Data are shown as mean ± SD; ns, not significant; one-way ANOVA for statistics.

Importantly, we confirmed that systemic expression of the tfLiveDrop reporter did not perturb normal physiology. Histopathological examination revealed no morphological abnormalities in heterozygous or homozygous reporter mice (Fig. 2A). Furthermore, despite the LD-targeting domain being derived from GPAT4, reporter expression did not induce systemic dyslipidemia, as shown by normal serum TG and TC levels (Fig. 2B), and showed no signs of aberrant tissue LDs accumulation, as assessed by PLIN2 immunostaining (Fig. 2C, D). Collectively, these data demonstrate that the tfLiveDrop mouse faithfully monitors lipophagic flux without perturbing endogenous lipid metabolism.

### Spatial profiling of basal lipophagic activity across systemic organs

**Fig. 3:**
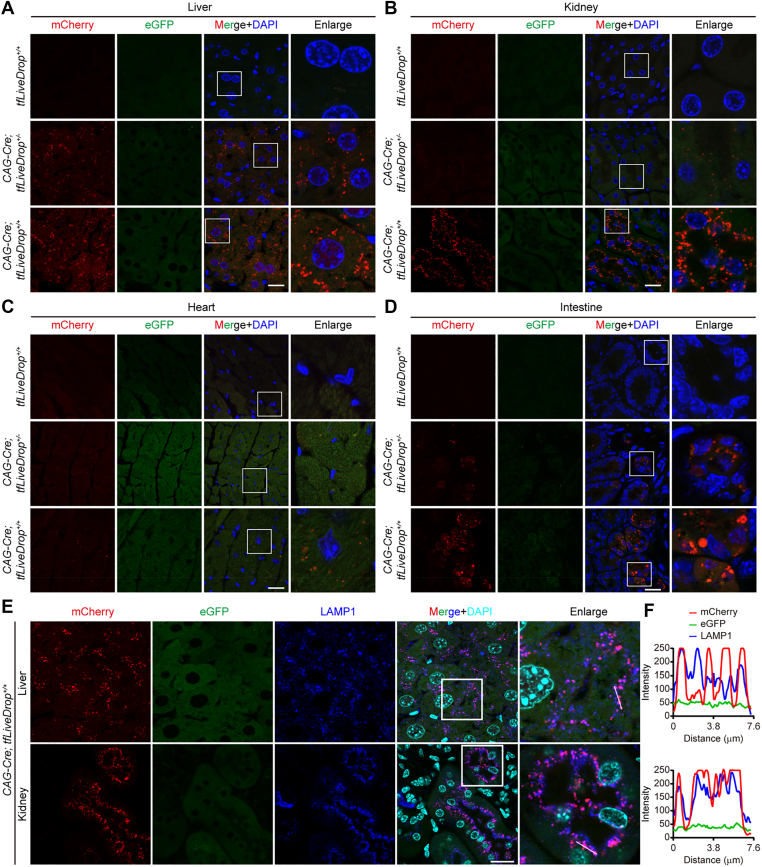
Spatial profiling of basal lipophagic activity across systemic organs. A–D: Confocal microscopy images of lipophagy activity in tissue sections (liver, kidney, heart, intestine) under basal conditions from male *tfLiveDrop*^+/+^, *CAG-Cre*; *tfLiveDrop*^+/^^−^, and *CAG-Cre*; *tfLiveDrop*^+/+^ mice.Scale bar: 20 μm. E: *Confocal microscopy images* of colocalization of tfLiveDrop signals with lysosomal marker LAMP1^+^ (*Blue*) in liver and kidney sections from *CAG-Cre;tfLiveDrop*^+/+^ mice. Scale bar: 20 μm. F: Plot profile of relative fluorescence intensities of mCherry, eGFP, and LAMP1 along the indicated path.

Having established and validated the tfLiveDrop reporter model, we next employed it to map basal lipophagic activity across multiple organs in vivo. We examined cryosections from adult male CAG-Cre;tfLiveDrop mice and their control littermates. As expected, no reporter fluorescence was detected in tissues from tfLiveDrop-only (Cre-negative) mice, confirming the tight control of expression by the Cre-loxP system. In reporter-positive mice, a distinct spatial pattern of basal lipophagic flux emerged (Fig. 3A–D, Supplemental Fig.S2A, B). The most prominent mCherry^+^eGFP^-^ puncta, indicative of ongoing lipophagy, were observed in hepatocytes (Fig. 3A). This aligns with the central role of liver in systemic lipid metabolism and was consistent with the predominant basal expression of the LD coat protein PLIN2 in this organ (Fig. 2C). Notably, clear mCherry^+^eGFP^-^ signals were also present within the intestinal crypts (Fig. 3D), the niche housing adult intestinal stem cells (22), suggesting a potential role for constitutive lipophagy in this compartment.

In contrast, basal lipophagic signals were minimal or undetectable in the kidney, heart, lung, and brain of heterozygous reporter mice (Fig. 3B, C, Supplemental Fig. S2A, B). Homozygous reporter mice showed intensified fluorescence in the liver and intestinal crypts, and sporadic signals in renal tubules and cardiomyocytes (Fig. 3, A–D). However, to preclude potential artifacts from long-term lysosomal fluorophore accumulation (23), all subsequent studies utilized heterozygous animals. Critically, the mCherry^+^eGFP^-^ puncta in reporter-positive tissues exhibited near-complete colocalization with the lysosomal marker LAMP1 (Fig. 3E, F), confirming that the tfLiveDrop signal specifically identifies LDs delivered to lysosomes and validating its utility for measuring lipophagic flux in vivo.

To confirm that the tfLiveDrop reporter is functional across sexes and to enable future studies on sex-dependent lipophagy regulation, we assessed reporter activity in female CAG-Cre;tfLiveDrop^+^^/^^−^ mice under short-term high-fat diet (HFD) feeding. Following three weeks of HFD to induce systemic lipid loading (Supplemental Fig. S3A, B), robust mCherry^+^eGFP^+^ and mCherry^+^eGFP^-^ puncta were observed in various organs, including the liver, kidney, heart, and intestine (Supplemental Fig. S3C, D). Consistent with our observations in male mice (Fig. 3), the liver exhibited the highest lipophagic activity, while the kidney showed moderate signals under lipid-loaded conditions. These data confirm that the tfLiveDrop reporter is fully functional in female mice and can be used to investigate sex-specific differences in lipophagy under both basal and stressed conditions.

### In vivo imaging reveals organ-specific impairment of lipophagic flux in T2D

**Fig. 4:**
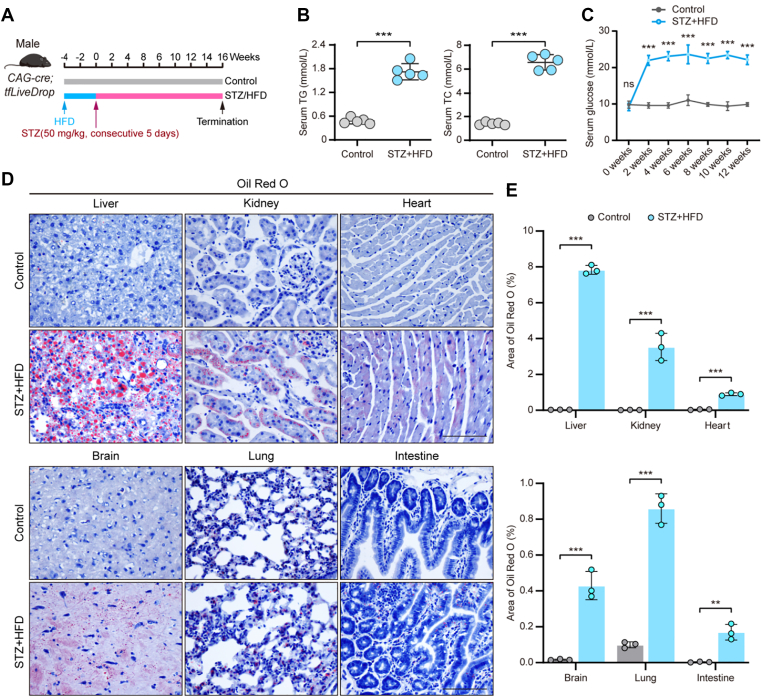
Establishment of a T2D model using *CAG-Cre*; tfLiveDrop reporter mice. A: Scheme for T2D mouse model induction in male *CAG-Cre*; *tfLiveDrop*^+/−^ mice, involving 1-month HFD feeding followed by 5-days streptozotocin (STZ) injections (50 mg/kg, i.p.), with termination at 16 weeks. B: Serum TG and TC levels in control and T2D *CAG-Cre; tfLiveDrop*^+/^^−^ mice (n = 5). C: Time-course serum glucose levels in control and T2D *CAG-Cre*; *tfLiveDrop*^+/^^−^ mice. D: Oil red O staining images of control and T2D *CAG-Cre*; *tfLiveDrop*^+/^^−^ mice (liver, kidney, heart, intestine, lung, brain). Scale bar: 50 μm. E: Quantification of Oil Red O^+^ areas. (n = 3). Data are shown as mean ± SD; ns, not significant; ∗∗*P* < 0.01, ∗∗∗*P* < 0.001; Student's *t* test for statistics.

To investigate the impact of systemic metabolic disease on lipophagic activity in vivo, we monitored lipophagic flux using male tfLiveDrop reporter mice in a murine model of T2D. *CAG-Cre; tfLiveDrop*^*+*^^/−^ reporter mice were subjected to a STZ/HFD regimen to induce T2D, a model known to cause systemic lipid metabolic disturbances (Fig. 4A). Compared with control mice, STZ/HFD-treated mice exhibited significantly elevated serum levels of TG, TC, and glucose, confirming successful model induction (Fig. 4B, C).

**Fig. 5:**
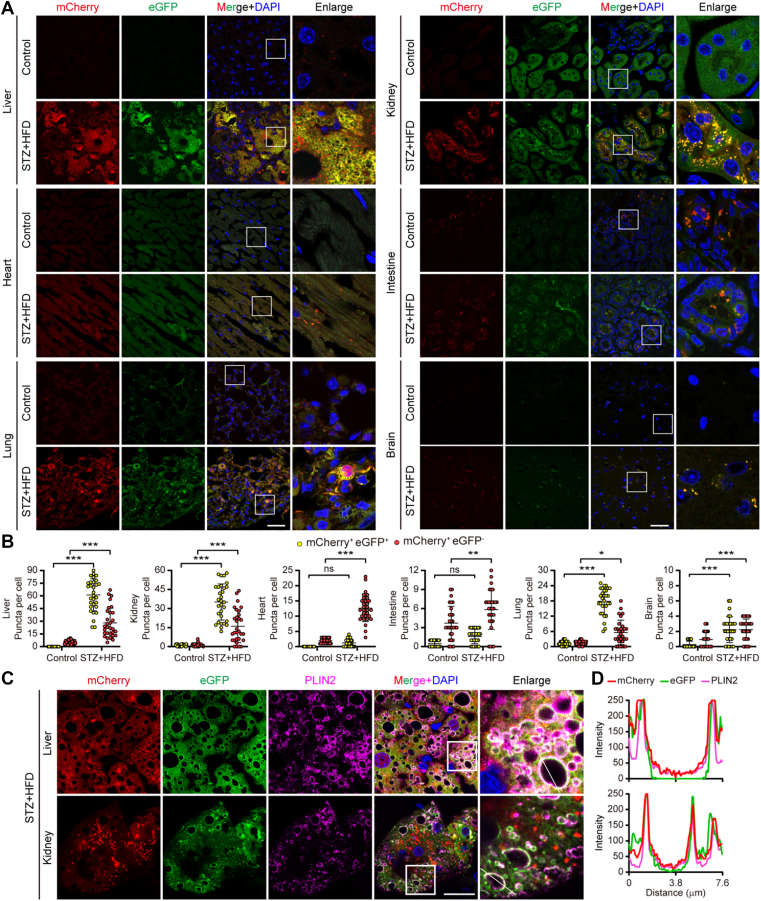
In vivo imaging reveals organ-specific impairment of lipophagic flux in T2D. A: *Confocal microscopy images* of lipophagy activity in tissue sections (kidney, heart, liver, intestine, lung, brain) from control and T2D *CAG-Cre*; *tfLiveDrop*^+/^^−^ male mice. Scale bar: 20 μm. B: Quantification of mCherry^+^eGFP^+^ (yellow, cytosolic) and mCherry^+^eGFP^-^ (red, lysosomal) puncta per cell in each organ (n = 30 cells per group). C: Confocal images of colocalization of tfLiveDrop signals with LD coat protein PLIN2 (magenta) in liver and kidney sections from *CAG-Cre; tfLiveDrop*^+^^/^^−^ mice. Scale bar: 20 μm. D: Plot profile of relative fluorescence intensities of mCherry, eGFP, and PLIN2 along the indicated path. Data are shown as mean ± SD; ns, not significant; ∗*P* < 0.05, ∗∗*P* < 0.01, ∗∗∗*P* < 0.001; one-way ANOVA for statistics.

Remarkably, confocal imaging of tissues from diabetic mice revealed a striking organ-specific dichotomy in lipophagic status (Fig. 5A, B). In major metabolic organs affected by diabetes, the liver and kidney, we observed a pronounced accumulation of mCherry^+^eGFP^+^ (yellow) puncta. This indicates a blockade of lipophagic flux, with LDs forming but not being efficiently delivered to lysosomes. In contrast, the heart, brain, lung, and small intestine predominantly exhibited mCherry^+^eGFP^-^ (red) puncta, signifying that lysosomal degradation of LDs remained operative in these organs under diabetic conditions. The lipophagic blockade in the liver and kidney was pathophysiologically consequential, as it strongly correlated with substantial lipid accumulation detected by Oil Red O staining (Fig. 4D, E). Furthermore, large mCherry^+^eGFP^+^ structures in these organs colocalized with PLIN2 (Fig. 5C, D), confirming the accumulation of undegraded LDs. These findings identify the liver and kidney as primary sites of defective lipophagic clearance in T2D, providing a mechanistic link to pathological lipid overload.

### Developmental dynamics of lipophagic flux in the kidney revealed by tfLiveDrop

**Fig. 6:**
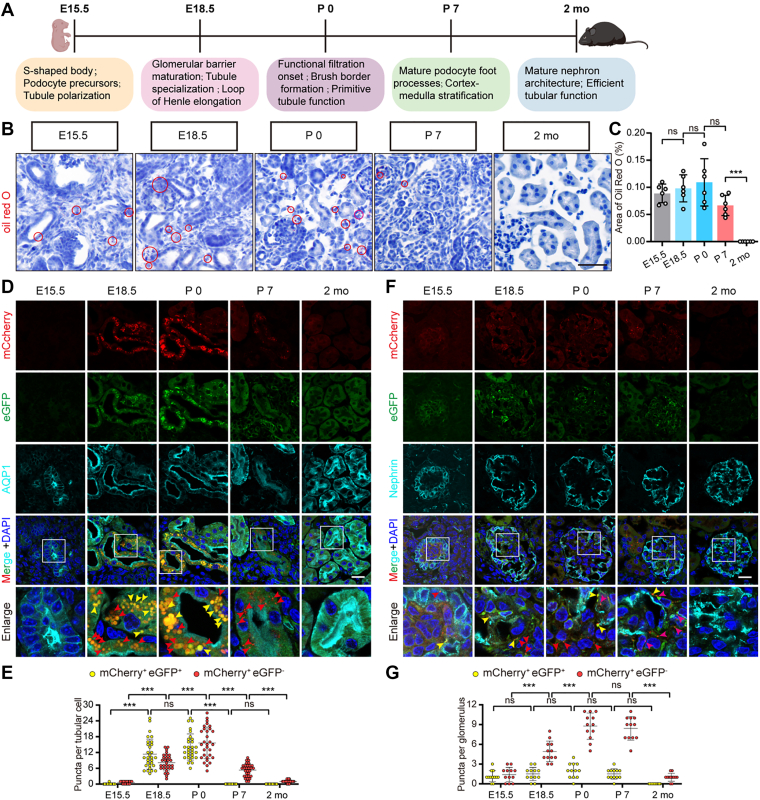
A transient perinatal wave of lipophagy coordinates renal metabolic maturation. A: Schematic timeline of significant stages in murine kidney development (E, embryonic day; P, postnatal day; mo, month), with key developmental events annotated. B: *Oil red* O staining images of kidney cryosections (red circles mark Oil Red O^+^ areas). C: Quantification of Oil Red O^+^ area in kidneys from *CAG-Cre*; *tfLiveDrop*^+/^^−^ mice at E15.5, E18.5, P0, P7, and 2 months (n = 6). Scale bar: 20 μm. D: Confocal images of tfLiveDrop signals in AQP1^+^ tubules (cyan) of kidney sections from *CAG-Cre*; *tfLiveDrop*^+/^^−^ mice at the indicated stages. Scale bar: 20 μm. E: Quantification of tfLiveDrop puncta in tubular cells (n = 30 cells per group). F: Confocal images of tfLiveDrop signals in nephrin^+^ (cyan) glomeruli at the indicated stages (yellow arrows, mCherry^+^eGFP^+^ puncta; red arrows, mCherry^+^eGFP^-^ puncta). Scale bar: 20 μm. G: Quantification of tfLiveDrop puncta per glomerulus (n = 12 glomeruli per group). Data are shown as mean ± SD; ns, not significant; ∗∗∗*P* < 0.001; one-way ANOVA for statistics.

Finally, we explored the role of lipophagy in renal development, a process involving a metabolic shift from glycolysis to fatty acid oxidation (24). We hypothesized that lipophagy facilitates lipid homeostasis during this transition and employed tfLiveDrop mice to resolve its spatiotemporal dynamics (Fig. 6A).

Our analysis uncovered a defined developmental window of transient lipid deposition followed by clearance. Oil Red O staining revealed lipid accumulation in the collecting duct at E15.5, with scattered signals in tubules and glomeruli at E18.5 and postnatal day 0 (P0). This lipid burden was subsequently resolved, dropping to undetectable levels from postnatal week 1 through adulthood (Fig. 6B, C, red circle), indicating a postnatal shift toward balanced lipid utilization. Strikingly, tfLiveDrop imaging demonstrated that this clearance coincides with a programmed wave of lipophagic activity. While no signal was detected at E15.5, lipophagy was robustly activated perinatally. At E18.5 and P0, abundant mCherry^+^eGFP^+^ (yellow) puncta were observed in AQP1^+^ (Aquaporin 1, a marker for renal proximal tubules) proximal tubules, indicating widespread LD engagement. Lipophagic flux peaked around postnatal week 1, marked by a dominant population of mCherry^+^eGFP^-^ (red) puncta, confirming efficient lysosomal degradation. By adulthood, this activity subsided to near-baseline levels (Fig. 6D, E). A similar, though markedly weaker, temporal trend was observed in nephrin^+^ (Nephrin, a marker for glomerular podocytes) glomeruli (Fig. 6F, G). These data demonstrate that lipophagy is developmentally programmed as a transient, adaptive response to clear perinatal lipid loads, exhibiting a spatial preference for proximal tubules consistent with their high metabolic demand.

## Discussion

In this study, we established the tfLiveDrop reporter mouse, a novel tool that bridges a critical gap in lipid research: the ability to visualize and quantify lipophagic flux in real-time within living organisms. By overcoming the technical limitations of static assays, this model not only validates the superior sensitivity of flux-based monitoring but also provides novel insights into the spatiotemporal regulation of lipophagy in health, metabolic disease, and organ development.

Growing evidence underscores the central role of lipophagy in maintaining lipid homeostasis and modulating the progression of metabolic disease (9, 13, 25). While significant strides have been made in in vitro detection (26, 27, 28), in vivo studies remain constrained by a critical methodological gap: the lack of tools to distinguish lipophagic flux from static LDs accumulation. Existing probes primarily label the lipid payload (28, 29), failing to capture dynamic turnover. This distinction is pathophysiologically vital because LD sequestration can be cytoprotective against lipotoxicity (29, 30, 31); thus, without tools to specifically monitor flux, it is impossible to determine when to target the pathway, and indiscriminate modulation of bulk autophagy risks disrupting this compensatory mechanism. To overcome this barrier, we developed the tfLiveDrop system by fusing the GPAT4-derived LiveDrop domain to a pH-sensitive tandem probe (32). This strategy has previously been validated as an effective in vitro lipophagy detection tool (33). Unlike full-length coat proteins (e.g., PLIN2) that may stabilize LDs (34), the LiveDrop domain targets LDs late in biogenesis without perturbing endogenous dynamics (19). This design enables a specific, ratiometric readout of flux by spectrally distinguishing cytosolic LDs (yellow) from those within acidic lysosomes (red). Furthermore, the Cre-LoxP-dependent architecture allows for precise, cell-type-specific tracing, offering spatiotemporal resolution unattainable by conventional bulk assays.

Lipophagy is a key regulatory process in lipid homeostasis (35). Our study demonstrates that under basal conditions, the spatial distribution of lipophagic flux closely parallels the patterns of bulk autophagy flux observed using LC3 tandem-fluorescent reporters (36), mitophagy flux revealed by the mito-QC model (15), ER-phagy flux traced by ER-phagy reporters (16), and recently reported lysophagy flux (37). The co-activation of these pathways in regenerative niches, such as intestinal crypts and liver zone 2 (38), suggests that metabolically active stem cell populations may constitutively engage selective autophagy to support rapid biomass turnover and quality control. Conversely, organs such as the heart, lung, and brain displayed negligible basal lipophagy. This supports the hypothesis that in quiescent tissues, lipophagy functions primarily as an “on-demand” stress response rather than a constitutive housekeeper (31). This dual nature, constitutive in metabolic hubs versus inducible in stable tissues, highlights the evolutionary efficiency of the pathway in resource allocation.

This spatiotemporal framework is strongly supported by our in vivo observations in a T2D model. For instance, the kidney, which shows minimal basal lipophagic activity, exhibited a marked induction of lipophagic flux under diabetic conditions, accompanied by a distinct flux blockade (i.e., accumulation of mCherry^+^eGFP^+^ yellow signals), suggesting that while LDs are targeted for lysosomal delivery, the terminal degradation step may be compromised—potentially due to impaired lysosomal function or autophagosome-lysosome fusion. This dynamic response illustrates the “stress-responsive” nature of lipophagy but also identifies a potential therapeutic window. Specifically, restoring lipophagic clearance capacity through precise modulation of the lipophagy pathway, rather than non-specific manipulation of bulk autophagy, may represent a targeted strategy to alleviate renal lipotoxicity without disrupting physiological LD-mediated cytoprotection. Thus, the spatiotemporal dynamics of lipophagy revealed by the tfLiveDrop model provide both a critical methodological tool and a conceptual framework for understanding the differential regulation of lipophagy in physiological maintenance versus pathological adaptation. These insights lay an important foundation for mechanistic studies of metabolic diseases and for the development of targeted therapeutic strategies.

Utilizing the tfLiveDrop reporter mice, our developmental studies delineated a previously unrecognized programmed wave of lipophagic activity in the kidney. Specifically, we observed a transient accumulation of lipids in the tubules during the perinatal period, which was subsequently resolved by robust lipophagy activation in the early postnatal stage, before returning to basal levels in adulthood. This dynamic pattern parallels the established metabolic switch in the developing kidney from a predominantly glycolytic to an oxidative state (39). It is noteworthy that these fluctuations were strictly confined to the tubules, with no comparable shifts observed in the glomeruli, consistent with the distinct metabolic profiles of renal tubules (oxidative phosphorylation-dependent) versus glomeruli (glycolysis-dominant) (40). Importantly, this phenomenon appears to be tightly coordinated with the ontogeny of renal metabolic enzymes. Because expression of key cytosolic lipolytic enzymes is minimal or absent during embryonic development (24), lipophagy may serve as a critical compensatory pathway for fatty acid degradation during this transitional phase, thereby supporting the energetic and membrane biogenesis demands of tissue formation. Postnatally, as mitochondrial fatty acid β-oxidation (FAO) capacity in renal TECs expands to meet heightened metabolic demands (41), the canonical lipolytic machinery is concurrently induced. The maturation of this efficient lipolysis-oxidation coupling likely drives the physiological downregulation of lipophagic flux observed in later stages. Collectively, these findings indicate that lipophagy is not merely a constitutive process but a tightly spatiotemporally regulated adaptive mechanism. Activated during a critical developmental window, it facilitates the timely clearance of transitional lipid loads generated during metabolic reprogramming. By doing so, lipophagy underpins the metabolic maturation and functional differentiation of TECs, ensuring proper nephron development and homeostasis. This discovery not only highlights a pivotal role for lipophagy in organogenesis but also broadens our understanding of how selective autophagy pathways contribute to mammalian developmental programming (42).

### The limitations of this study

Despite the powerful tools provided by this study, several limitations warrant consideration. Primarily, our current analysis provides a snapshot view of lipophagic flux under static conditions. Given that lipophagic flux is a highly dynamic process, future longitudinal tracking will be indispensable to delineate the precise spatiotemporal onset of flux impairment during chronic disease progression. Furthermore, while the ubiquitous Cre-mediated expression of our reporter provided a systemic overview, it lacks the resolution to distinguish cell-type-specific metabolic signatures. The implementation of lineage-specific drivers, such as Ksp-Cre for TECs, will facilitate a more granular mapping of how individual cell populations contribute to the global homeostatic decline.

Notably, our experimental cohorts focused predominantly on male mice, a decision informed by the established sexual dimorphism in metabolic susceptibility and ischemic sensitivity (43, 44, 45). Our preliminary assessments in 8-week-old female CAG-Cre;tfLiveDrop mice on a short-term HFD confirmed the reporter's functionality across sexes. However, a comprehensive long-term comparison remains to be made. Given that sexually dimorphic liver lipid metabolism may influence lipophagic rates, fully quantifying these divergences represents a compelling frontier for future investigation.

Finally, as the LiveDrop domain derived from GPAT4, targets the general LD surface, the current reporter primarily monitors bulk LD-lysosome delivery. Our results demonstrate that both genetic ablation (*Atg5*-KO) and pharmacological inhibition (3-MA and BafA1) significantly impair this flux (Fig. 1F, L), suggesting that both macroautophagy and microautophagy contribute, consistent with previous reports (14, 46). However, BafA1 treatment resulted in a more pronounced accumulation of mCherry^+^eGFP^+^ LDs compared to 3-MA treatment. Since BafA1 blocks autophagosome-lysosome fusion and impairs lysosomal function (including microlipophagy), while 3-MA primarily inhibits autophagosome formation, the greater effect of BafA1 may reflect a more critical role for lysosomal function in LD degradation in renal TECs. Dissecting the relative contributions of macrolipophagy and microlipophagy will require future studies using receptor-specific genetic ablations.

In summary, the tfLiveDrop mouse represents a robust platform for dissecting the in vivo dynamics of lipid metabolism. By revealing the precise spatiotemporal patterns of lipophagy, this tool fundamentally advances our understanding of how cells handle lipid stress in diabetes and how organs remodel lipid stores during development, paving the way for targeted therapeutic interventions.

## Data Availability

The datasets generated during and/or analyzed during the current study are available from the corresponding author upon reasonable request.

## Supplemental Data

The Supplemental Data file provides the one figure supporting the study's findings.This article contains supplemental data.

## Conflict of Interest

The authors declare that they do not have any conflicts of interest with the content of this article.

## Supplemental Data

Supplemental Figures

Supplemental Raw Data
