# Supplementary material for: In vivo visualization of lipophagy dynamics using the tfLiveDrop reporter mice
Source: J Lipid Res. 2026 Apr 2;67(5):101033. doi: 10.1016/j.jlr.2026.101033 (PMC13137028; doi:10.1016/j.jlr.2026.101033)
Supplement: Supplemental Figures [file mmc1.docx]

**Supplementary Figures**

**In vivo visualization of lipophagy dynamics using the tfLiveDrop reporter mice**

Siqiao Gong^1^**^†^**, Qiaofei Zhang^1^**^†^**, Hongluan Wu^1^**^†^**, Xiaocui Chen^1^, Lijing Liu^1^,Yongming Chen^1^, ZeSen Feng^1^, Shangmei Li^1^, Hongyong Su^1^, Jiansong Qi^1^, Jixin Tang^1^, Zhennan Ye^1^, Chen Yang^1*^, Huafeng Liu^1*^

^1^Department of Nephrology, National Clinical Key Specialty Construction Program (2023), Institute of Nephrology, Guangdong Provincial Key Laboratory of Autophagy and Major Chronic Non-communicable Diseases, Key Laboratory of Prevention and Management of Chronic Kidney Disease of Zhanjiang City, Affiliated Hospital of Guangdong Medical University, Zhanjiang, China.


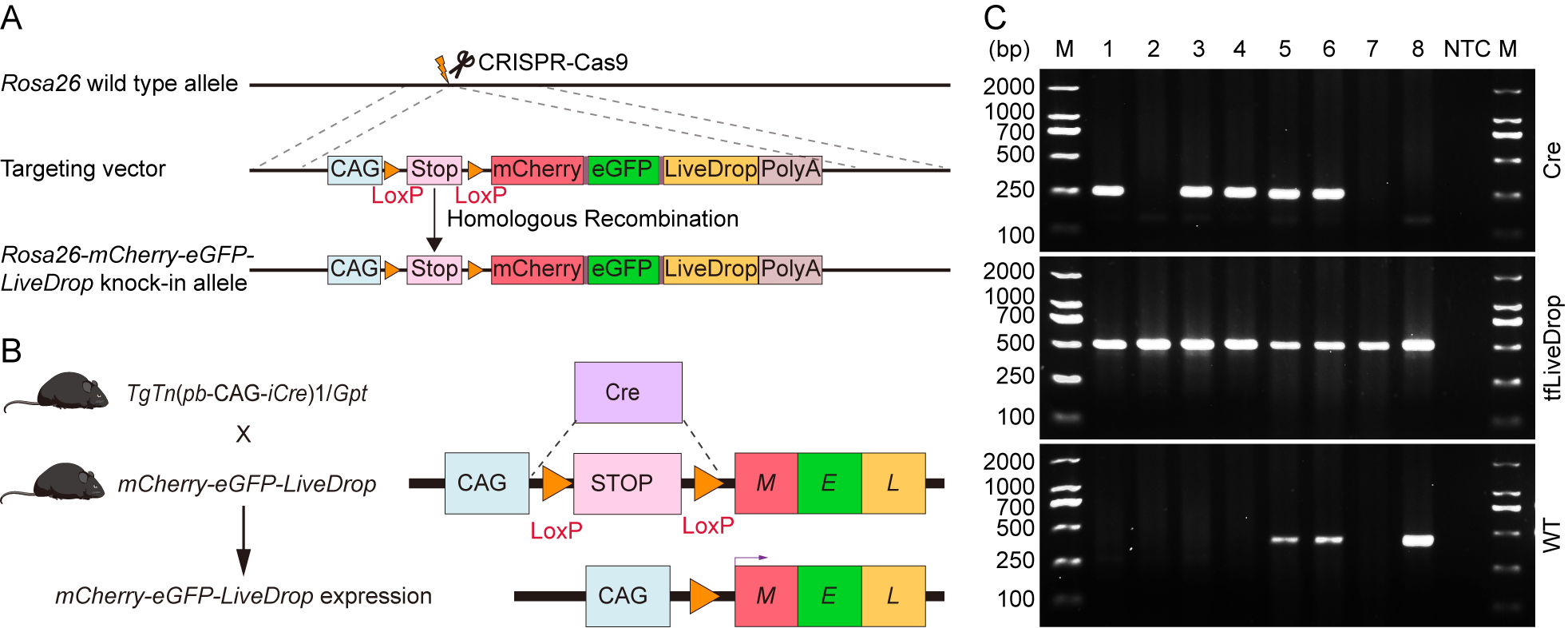


**Supplementary Fig. S1. Design and generation of the tfLiveDrop reporter mice.** Related to Figure 1. (**A**) Strategy for generating the *Rosa26*-targeted *tfLiveDrop* conditional knock-in mouse. (**B**) Breeding scheme to generate *CAG-Cre*; *tfLiveDrop* reporter mice. (**C**) Genotyping verification by PCR analysis of tail genomic DNA. Lanes 1-8: individual mice; M: molecular weight marker; NTC: no template control (nuclease-free water). Genotypes: Lanes 1, 3, 4: *CAG-Cre*; *tfLiveDrop* homozygous; Lanes 5, 6: *CAG-Cre*; *tfLiveDrop* heterozygous; Lanes 2, 7: *tfLiveDrop* homozygous; Lane 8: *tfLiveDrop* heterozygous.

**
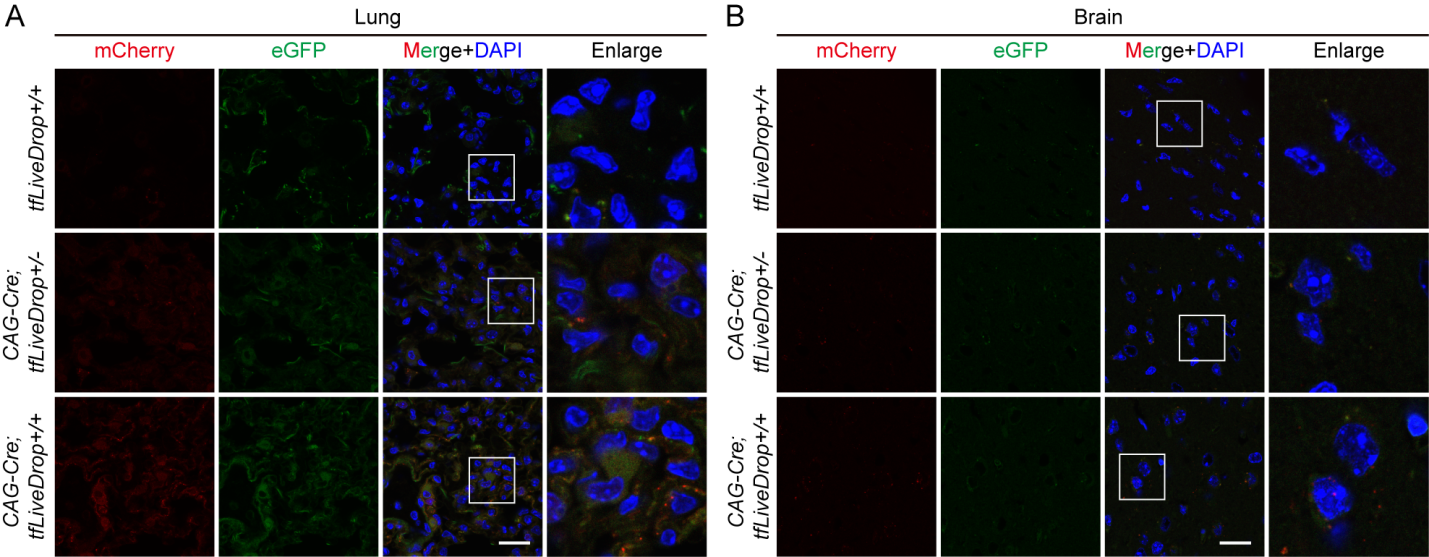
**

**Supplementary Fig. S2. Basal lipophagic activity in lung and brain.** Related to Figure 3. (**A,B**) Confocal microscopy images of lipophagy activity in lung and brain sections under basal condition from male tfLiveDrop,CAG-Cre; tfLiveDrop^+/-^ and CAG-Cre; tfLiveDrop^+/+^ mice. Scale bar: 20 μm.


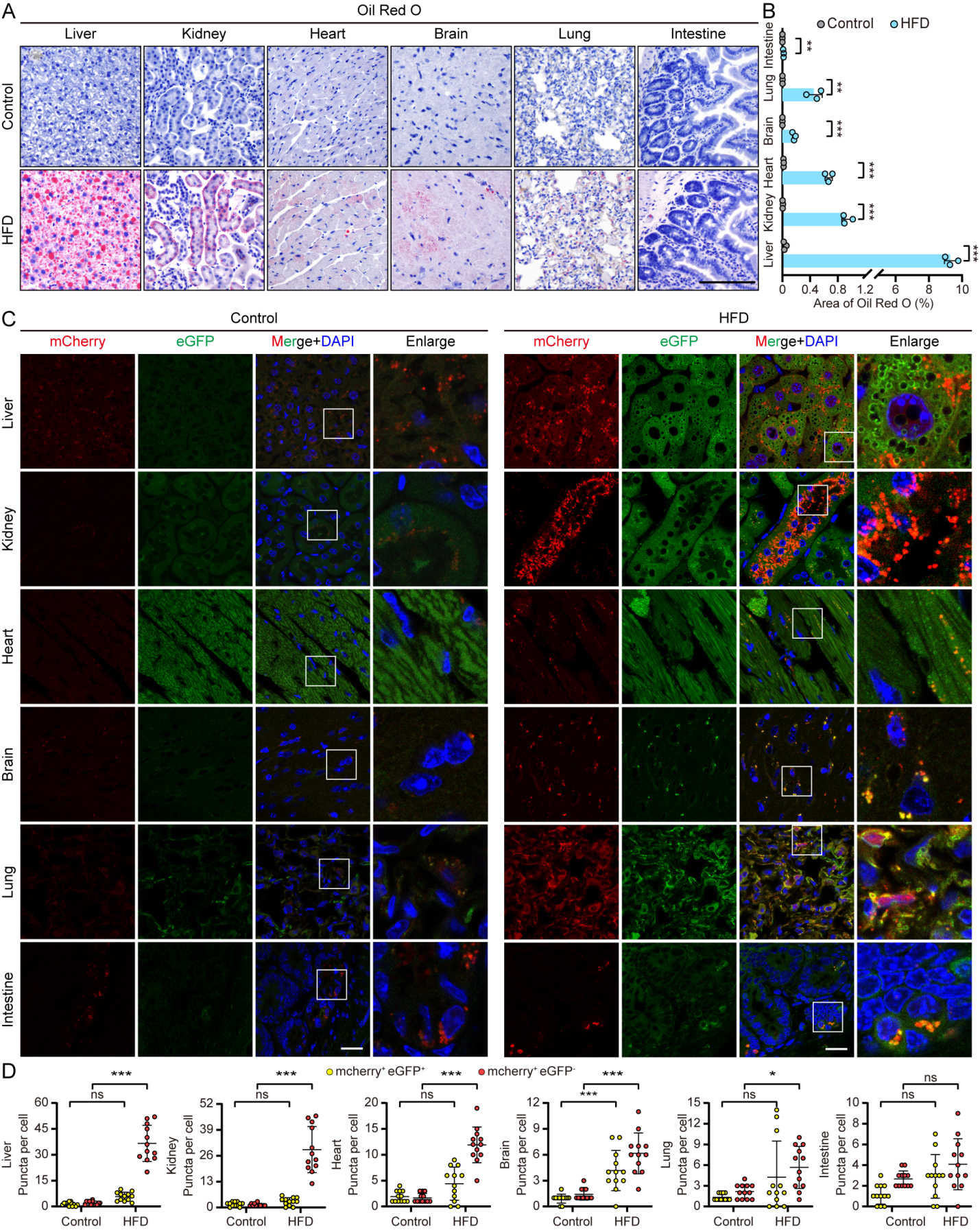


**Supplementary Fig. S3. Lipophagic flux in various organs of female CAG-Cre;tfLiveDrop^+/-^ mice under short-term HFD feeding.** (**A**) Oil Red O staining in systemic organs (Liver, Kidney, Heart, Brain, Lung, and Intestine) from control and HFD-fed female CAG-Cre;tfLiveDrop^+/-^ mice. Scale bar: 50 μm. (**B**) Quantification of Oil Red O⁺ areas (n = 3). (**C**) Confocal microscopy images of lipophagy activity in systemic organs sections under short-term HFD feeding condition from female CAG-Cre;tfLiveDrop^+/-^ mice. Scale bar: 20 μm. (**D**) Quantification of mCherry^+^eGFP^+^ and mCherry^+^eGFP^-^ puncta per cell in each organ (n = 12 cells per group). Data are shown as mean ± SD; ns, not significant, **P* < 0.05, ***P* < 0.01, ****P* < 0.001; Student’s *t*-test for (B), and one-way ANOVA for (D).

**Movie S1.** **Time-lapse imaging of lipophagic flux.** Corresponds to still frames from the panel in Figure 1K. Primary renal TECs isolated from CAG-Cre;tfLiveDrop+/- male mice were pulsed with 0.2 mM FFA for 24 h and subsequently chased under starvation conditions for 12 h. The movie captures the dynamic transition of a dual-fluorescent LD (mCherry⁺eGFP⁺) into a lysosomal compartment (labeled with LysoTracker Deep Red, blue), resulting in the quenching of eGFP and the persistence of mCherry+ only fluorescence, indicated by the white box (ROI 1A). Movie represents 8 min 2.38 sec of elapsed time with frames captured every 19.3 sec. Playback is at 3 frames/sec. Scale bar: 10 μm.
