# Supplementary material for: In vivo visualization of lipophagy dynamics using the tfLiveDrop reporter mice
Source: J Lipid Res. 2026 Apr 2;67(5):101033. doi: 10.1016/j.jlr.2026.101033 (PMC13137028; doi:10.1016/j.jlr.2026.101033)
Supplement: Supplemental Raw Data [file mmc2.docx]

**Supplementary Raw Data**

**In vivo visualization of lipophagy dynamics using the tfLiveDrop reporter mice**

Siqiao Gong^1^**^†^**, Qiaofei Zhang^1^**^†^**, Hongluan Wu^1^**^†^**, Xiaocui Chen^1^, Lijing Liu^1^,Yongming Chen^1^, ZeSen Feng^1^, Shangmei Li^1^, Hongyong Su^1^, Jiansong Qi^1^, Jixin Tang^1^, Zhennan Ye^1^, Chen Yang^1*^, Huafeng Liu^1*^

^1^Department of Nephrology, National Clinical Key Specialty Construction Program (2023), Institute of Nephrology, Guangdong Provincial Key Laboratory of Autophagy and Major Chronic Non-communicable Diseases, Key Laboratory of Prevention and Management of Chronic Kidney Disease of Zhanjiang City, Affiliated Hospital of Guangdong Medical University, Zhanjiang, China.

**Fig 1D**

**
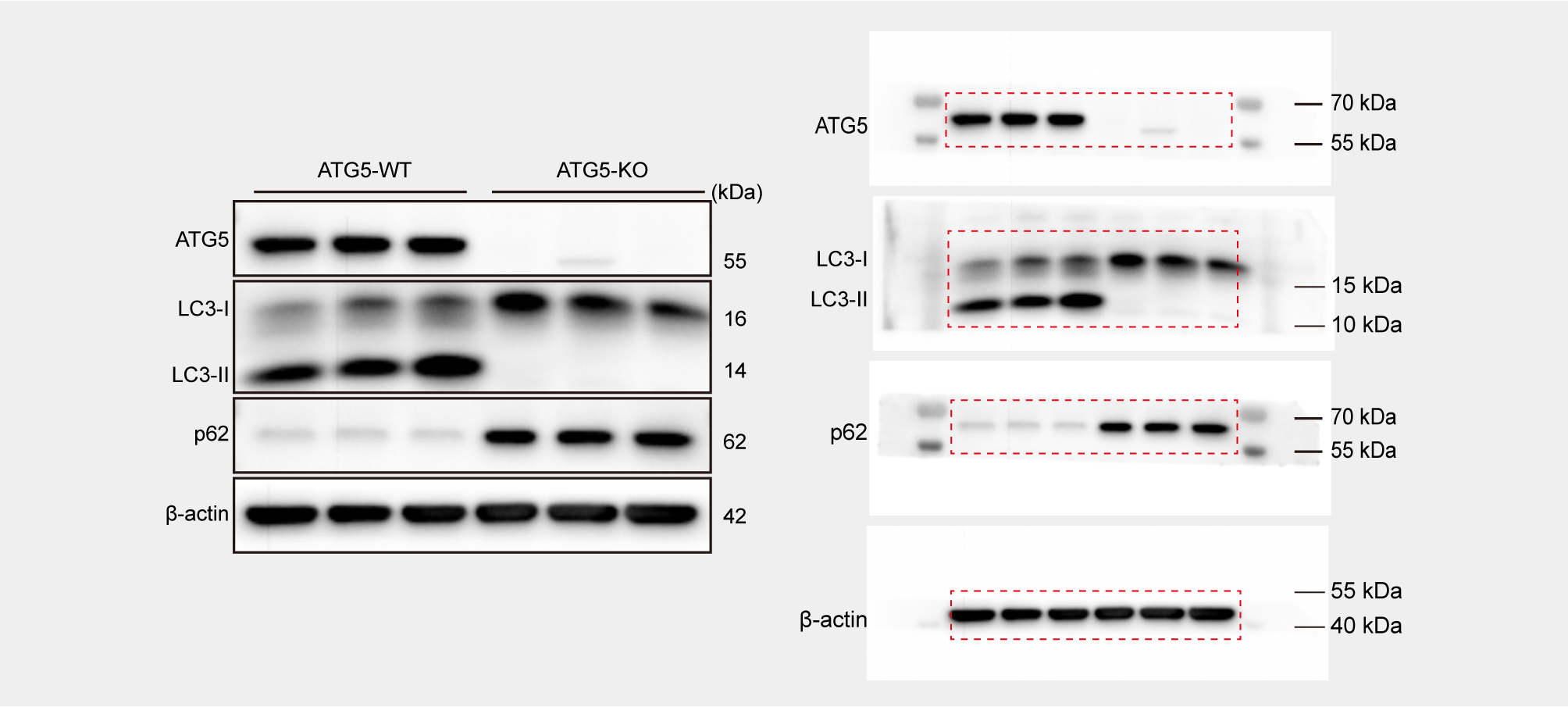
**

**Fig S1C**

**
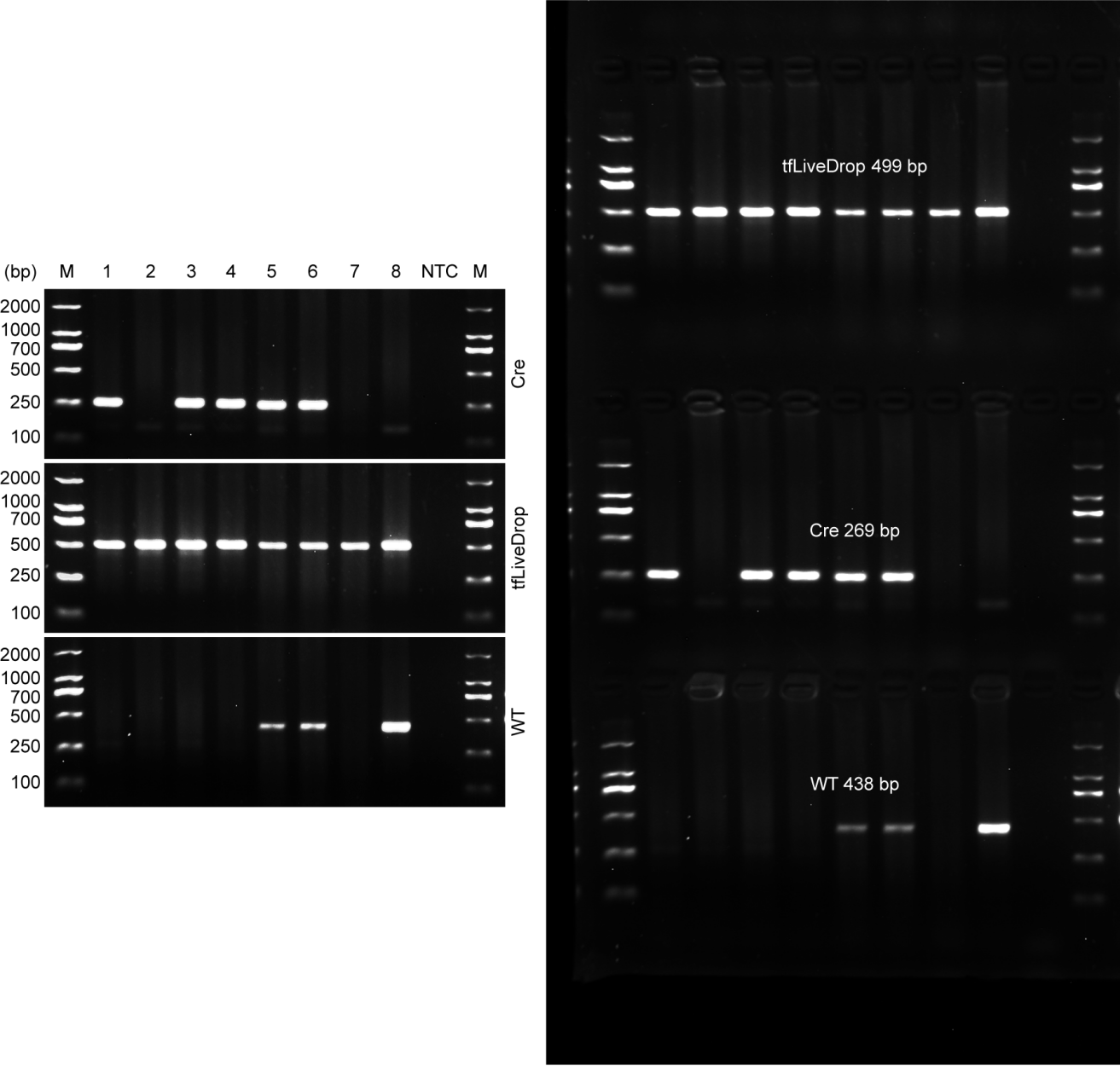
**
